# Preoperatively predicting failure to achieve the minimum clinically important difference and substantial clinical benefit for total knee arthroplasty patients using machine learning

**DOI:** 10.1186/s43019-025-00289-y

**Published:** 2025-09-10

**Authors:** Jaeyoung Park, Emilie N. Miley, Xiang Zhong, Chancellor F. Gray

**Affiliations:** 1 School of Global Health Management and Informatics, University of Central Florida, Orlando, FL 32801 USA; 2 Institute of Sports Sciences and Medicine, Department of Health, Nutrition and Food Sciences, Florida State University, Tallahassee, FL 32306 USA; 3 Tallahassee Orthopedic Clinic, Tallahassee, FL 32308 USA; 4 Department of Industrial and Systems Engineering, University of Florida, Gainesville, FL 32603 USA; 5 Florida Orthopaedic Institute, Gainesville, FL 32607 USA

**Keywords:** Patient-reported outcome measures, Minimal clinical important difference, Substantial clinical benefit, Total knee arthroplasty, Machine learning, Risk factor analysis

## Abstract

**Background:**

A clear understanding of minimal clinically important difference (MCID) and substantial clinical benefit (SCB) is essential for effectively implementing patient-reported outcome measurements (PROMs) as a performance measure for total knee arthroplasty (TKA). Since not achieving MCID and SCB may reflect suboptimal surgical benefit, the primary aim of this study was to use machine learning to predict patients who may not achieve the threshold-based outcomes (i.e., MCID and SCB) on the Knee Injury and Osteoarthritis Outcome Score for Joint Replacement (KOOS JR) following TKA.

**Methods:**

Data from 1064 patients who underwent TKA at a single academic medical center between 2016 and 2022 contained 81 preoperative variables, including routinely collected measures and PROMs (KOOS JR and Patient-Reported Outcomes Measurement Information Systems [PROMIS-10]). Several machine-learning models were developed, which include penalized logistic regression as a linear model, support vector machine with polynomial and radial kernels as nonlinear models, and random forest and extreme gradient boosting as nonparametric models. These models predicted both distribution- and anchor-based MCIDs and SCB. In addition, logistic regression models were used to identify relevant risk factors for failing to meet these thresholds.

**Results:**

The random forest models and the penalized logistic regression models achieved acceptable area under the receiver operating characteristic curve (AUC) close to or above 0.7 for all the outcomes. Furthermore, the logistic regression models identified shared risk factors for the three outcomes: preoperative PROMs (i.e., KOOS JR score, PROMIS-10 global physical T-score, and PROMIS-10 general mental health), antidepressant medication history, age, and Kellgren–Lawrence grade.

**Conclusions:**

Machine-learning models were able to identify patients at risk of failure to achieve the threshold-based metrics and relevant preoperative factors. As such, these models may be used to both improve shared decision-making and help create risk-stratification tools to improve quality assessment of surgical outcomes.

**Supplementary Information:**

The online version contains supplementary material available at 10.1186/s43019-025-00289-y.

## Introduction

Patient-reported outcome measures (PROMs) are increasingly collected to monitor the impact of care episode delivery [1, 2]. These PROMs are intended to capture patients’ perspectives on their health conditions while complementing the evaluations traditionally made by healthcare providers [1, 3, 4]. Further, they provide a quantitative assessment of the effect on the healthcare intervention, theoretically allowing for comparisons across interventions, providers, sites of care, and even across patient factors. By capturing and comparing pre- and post-intervention PROM scores, clinicians can assess the patient’s perspective of the intervention and gauge a level of improvement [3, 5–9]. To aid in grading the impact of the intervention, a threshold-based outcome (i.e., whether the change in PROMs surpasses a specific level) is often used to evaluate the results [10].

Currently, several options exist to determine these thresholds. The minimal clinically important difference (MCID) [11], first defined by Jaeschke and colleagues in 1989, is intended to represent the smallest score difference that patients recognize as beneficial. Secondarily, the substantial clinical benefit (SCB) [13, 14], originally created by Glassman et al. and adopted for use in the total knee arthroplasty (TKA) population by Lyman et al. in 2017, has recently gained more visibility as it has been selected for mandatory reporting purposes [12]. The SCB represents the change where patients experience a substantial (i.e., rather than minimally detectable) improvement, serving as a benchmark for defining clinical success [13], especially in arthroplasty [14, 15]. Emphasizing the valued use of PROMs, the Centers for Medicare and Medicaid Services (CMS) has defined achievement of the SCB as a quality measure in arthroplasty that will be tracked and will influence reimbursement, intending to encourage high-quality care [16, 17].

The central role of PROMs in shared decision-making and as a key element of patient-centered care, along with their potential role in reimbursement determinations, makes understanding these instruments a core competency for the modern arthroplasty surgeon [18]. However, it is evident that a knowledge gap remains in how best to score and interpret these results. Currently, few standardized methods have been used to assign these thresholds. Moreover, the current risk adjustment method may be ineffective if CMS plans to use SCB to deem an encounter a success or failure [12, 16]; moreover, patient factors that predispose patients to either success or failure of the intervention are not routinely incorporated into the scoring system. This places some care episodes at substantial risk for failure to meet these thresholds, despite the high-quality decision-making and surgical intervention.

Currently, machine learning is widely used in the medical field to predict adverse outcomes and identify the factors driving these outcomes [19, 20]. Machine-learning models can process the vast amounts of data collected in healthcare, sparing clinicians from having to analyze all available information manually. In addition, these models have the potential to uncover risk factors that might not be identified using traditional models with fewer covariates [20, 21]. In addition, machine learning can assist clinicians in pinpointing high-risk patients prior to interventions to plan these interventions more effectively. Given that PROM-based metrics are now used to measure the quality of care, using machine learning to predict preoperative factors could enhance outcomes throughout a TKA intervention, as machine learning has demonstrated strong performance throughout other health outcomes, especially in arthroplasty, such as readmission [22, 23] and length of stay [24, 25].

The primary aim of this study was to use machine learning to predict patients who may not achieve the threshold-based outcomes (i.e., MCID and SCB) on the Knee Injury and Osteoarthritis Outcome Score for Joint Replacement (KOOS JR) [26] in a consecutive population of patients undergoing a total knee arthroplasty (TKA) at a single institution. In addition, we sought to identify relevant risk factors associated with failure to achieve the threshold-based outcomes available preoperatively to aid in both shared decision-making and potential risk stratification.

## Materials and methods

### Patient population

**Fig. 1:**
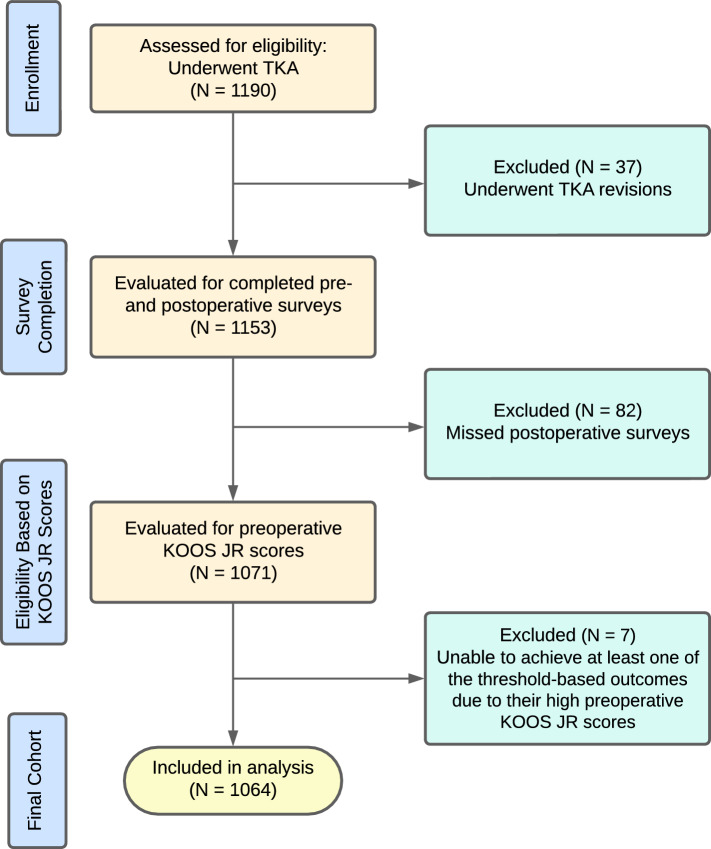
Study cohort identification. *KOOS JR* Knee Disability and Osteoarthritis Outcome Score Joint Replacement, *TKA* total knee arthroplasty

After institutional review board approval, data were retrospectively collected from the electronic health records (Epic Systems, Verona, WI, USA) for patients who underwent a primary TKA (CPT: 27447) at a single tertiary care center between January 2016 and July 2022. Patients were included in the study if they (1) had a primary TKA procedure performed and (2) had both preoperative (i.e., within 30 days) and 1-year postoperative (i.e., range 270–365 days) PROM scores specific to the KOOS JR and the Patient-Reported Outcomes Measurement Information Systems (PROMIS-10) [27]. For those patients who received multiple TKAs, only the first surgery was deemed as an index surgery and included. In addition, patients who were unable to achieve MCID and SCB (i.e., based on a preoperative KOOS JR score) were excluded owing to the inability to reach the change score per the definition [28]. This study cohort identification is shown in Fig. 1.

### Patient-reported outcome measures

PROMs collected were specific to the PROMIS-10 and the KOOS JR. The PROMIS-10, version 1.2, is a ten-item general health PROM that assesses patients’ overall general physical health (i.e., global physical T-score) and mental health (i.e., global mental T-score). The PROMIS-10 items are scored on a range from 1 (i.e., “poor,” “not at all,” “always,” and “very severe”) to 5 (i.e., “none,” “completely,” and “never”), which is then summed and transformed to the T-score, ranging from 16.2 (i.e., worse overall physical health) to 67.7 (i.e., best overall physical health) [29].

The KOOS JR is a region-specific and disease-specific instrument aimed at measuring knee pain and function. The KOOS JR includes seven items scored from 0 (i.e., none) to 4 (i.e., extreme). The score is then summed on a scale from 0 to 28, then inversely transposed to an interval score of 0–100, where 0 is indicative of total knee disability, and 100 is indicative of perfect knee health [26]. In this study, all the survey-related items were considered as continuous variables [30].

### Threshold-based outcomes

Specific outcomes of interest included the change in KOOS JR scores from the preoperative visit to postoperative visit (i.e., delta), not achieving two different MCIDs (i.e., anchor-based MCID and distribution-based MCID), and not achieving the SCB [10, 31, 32]. Anchor-based MCID and SCB rely on an anchor question that is determined by comparing the score change with corresponding responses to this question within the intended study population [31]. In 2017, Lyman et al. used the quality of life question from the Hospital for Special Surgery satisfaction survey as an anchor to derive both the anchor-based MCID and SCB [10]; this calculation was based on the comparison of the answers (i.e., those who reported “moderate improvement” [MCID] and those who reported “more improvement than I ever dreamed possible” and “great improvement” [SCB]) [10]. Several logistic regression models were constructed with each dichotomous independent variable based on KOOS JR scores and a dichotomous dependent variable based on the anchor question to identify the threshold for KOOS JR scores [10]. As an anchor question was not routinely collected, the present study adopted the anchor-based MCID threshold (i.e., 13.6 points) and the SCB threshold (i.e., 20 points) from the previous study by Lyman et al. [10]. In contrast, the distribution-based MCID is typically calculated from the statistical distribution of the population of interest [31]. Even though this value is considered less validated than the anchor-based MCID [10], the distribution-based MCID is easily accessible. Using the standard approach (i.e., 0.5 × the standard deviation of the difference in KOOS JR scores) [10], the distribution-based MCID was calculated to be 9.6 points within this study population.

### Covariates

Covariates included in the analyses were comparable to those used in similar existing studies assessing outcomes related to PROMs [28, 33–35], along with additional relevant covariates accessible in the electronic health record (EHR) system for knowledge discovery. Covariates included in the set available at the time of TKA decision-making and the following categories were considered: (1) demographic (e.g., age and sex) and socioeconomic status, (2) preoperative care (e.g., body mass index [BMI] and Charlson Comorbidity Index [CCI]), (3) comorbidities and medical histories documented within 90 days of the index procedure, (4) medications documented within 90 days of the index procedure, and (5) preoperative survey. The covariates are presented in Table 1 for preoperative survey and Supplementary Material S1, with key highlights presented here. The Kellgren–Lawrence (KL) score, which indicates the presence and severity of knee radiographic osteoarthritis, and American Society of Anesthesiologists (ASA) physical status classification [36, 37] were collected during preoperative care. In addition, the age variable was categorized on the basis of quartiles (i.e., 18–66, 67–71, 72–77, and ≥ 78 years) [38]; BMI was based on standard categories (i.e., < 25.0, 25.0–29.9, 30.0–39.9, and ≥ 40 kg/m^2^) [39].

### Statistical analysis

Descriptive analyses were first conducted, including (1) examining the univariate association of each variable with the delta and (2) visualizing the preoperative scores (i.e., KOOS JR score and PROMIS-10 global physical health T-score) and the outcomes. Significant variables were identified using *t*-test for binary variables, analysis of variance (ANOVA) for categorical variables, and Pearson’s correlation test for continuous variables with an alpha level of *P* < 0.05.

Subsequently, machine-learning models were developed to predict the failure to achieve the MCIDs and SCB, respectively. Analyses were separated into prediction evaluation and risk factor identification. Prediction evaluation demonstrated how well models predict the outcomes within the data, while risk factor identification demonstrated what preoperative variables should be considered. For model evaluation, the entire dataset was split into a training set (i.e., for model building) and a test set (i.e., for assessments) in a 4:1 ratio [40]. Machine-learning models included penalized logistic regression (PLR) as a linear model, support vector machine (SVM) with polynomial and radial kernels as nonlinear models, and random forest (RF) and extreme gradient boosting (XGBoost) as nonparametric models. Deep-learning models and class balancing techniques were not considered for this analysis owing to the limited sample size [41, 42].

Regarding model specification, we used the least absolute shrinkage and selection operator (LASSO) for PLR models, tuning the penalty weight (*λ*) through cross-validation [43]. We built SVM models using two different kernels (i.e., polynomial and radial kernels), tuning the cost parameter (i.e., ranging from 1 to 5) [44]. In RF models, we fine-tuned the number of random variables selected at each split (3, 4, and 5) and the number of trees to grow (50, 100, 150, and 200) [45]. For the XGBoost models, optimal hyperparameters were identified for the L2 regularization term on weights (*λ*, tested at 0.01, 0.1, 0.5, 1, and 2) and the maximum tree depth, which ranged from 3 to 8 [46]. For hyperparameter tuning, we selected the hyperparameter that resulted in the smallest cross-validation error.

The model performances were measured using area under the receiver operating characteristic curve (AUC) and F1 score. The AUC assesses a model’s ability to distinguish between high-risk and low-risk patients [47], whereas the F1 score considers both recall (i.e., accounting for false negatives) and precision (i.e., accounting for false positives), while robustly evaluating machine-learning models for imbalanced outcomes [48]. Since false positives can lead to unnecessary care and false negatives may result in missed treatment opportunities, reporting the F1 score is essential for evaluating the performance of machine-learning models. Unlike the AUC, the F1 score requires a cutoff that divides estimated probabilities into two classes. We obtained the optimal cutoff that maximized the F1 score for the training set and applied the same for the test set [49]. In addition, the model performances were compared with the baselines of the AUC and the F1 score, which were obtained by randomly assigning outcomes with equal probability [50]. Being close to the baseline, such as the fixed value of 0.5 for AUC, indicates no improvement [50, 51]. To calculate the 95% confidence interval (CI) of model performances, the evaluation procedure was repeated 100 times [45].

For risk factor identification, logistic regression model was selected, as the linear models performed comparably well in the data to the RF models (Table 2). According to Occam’s razor [52], simpler models such as linear models are less susceptible to overfitting than complex models such as RF. In addition, the logistic regression model uniquely provides both directions and magnitudes of associations. To identify risk factors, a stepwise variable selection using Akaike information criterion from 81 variables was performed first to prevent overfitting and ensure the models remain generalizable [53]. Then, a logistic regression model was developed including the selected variables for each outcome and considered variables with *P* < 0.05 as significant [54]. Significant variables that shared commonality for the three outcomes were compared in terms of their direction and magnitude. In addition, an odds ratio (OR) < 1 indicates higher likelihood of achieving an MCID or an SCB, while an OR > 1 indicates a lower likelihood [55]. For the sanity check, a linear regression model with the same variable selection method was built [56]. All statistical analyses were conducted in R, version 4.2 [57], with the following packages: mice [58], glmnet [43], e1707 [59], randomForest [60], xgboost [46], and PROC [61].

## Results

We identified 1064 patients who completed both preoperative and postoperative surveys. The cohort was divided into the following four age groups, each representing a quartile of the population: 18–66 years (24.9%), 67–71 years (21.1%), 72–77 years (28.3%), and 78 years or older (25.8%). The average preoperative KOOS JR score significantly differed between age groups (F[3, 1060] = 12.1, *P* < 0.001), with overall scores increasing across age groups (18–66 = 41.1, 67–71 = 45.7, 72–77 = 46.4, and ≥ 78 = 47.9). There were more female patients (*N* = 659, 61.9%) than male patients (*N* = 405, 38.1%), and the population was predominantly White (*N* = 679, 63.8%; Supplementary Table S1).

### Threshold outcomes and change score

Correlation values between the delta and the survey-related variables are presented in Table 1. For all the KOOS JR individual survey items, patients who responded “extreme” were more likely to achieve significant improvement (*r* = 0.322–0.406, *P* < 0.001). Conversely, mixed correlations were observed across the PROMIS-10 items: the PROMIS-10 global health physical T-score was negatively correlated with total change (*r* = −0.161, *P* < 0.001), whereas Global03 (i.e., “In general, how would you rate your physical health?”) was not (*r* = 0.003, *P* = 0.93). Nonetheless, similar to the KOOS JR, responses of “poor” on physical health items were consistently weakly correlated with an increase in the KOOS JR score (*r* = −0.266 to −0.104, *P* < 0.001; Global06–08 in Table 1). Patients who responded “excellent” to Global04 (i.e., “In general, how would you rate your mental health, including your mood and your ability to think?”) were likely to experience an increase in their KOOS JR scores (*r* = 0.084; *P* = 0.006). In contrast, a higher global mental health T-score was not significantly associated with the score difference (*r* = 0.024, *P* = 0.433).

**Table 1:** Univariate relationships between the change in KOOS JR scores and individual survey variables

Covariates	Pearson’s correlation coefficient (*r*)	*P*-value
KOOS JR^†^		
KOOS JR score	−0.530	< 0.001
Question (1)—Stiffness	0.372	< 0.001
Question (2)—Twisting	0.322	< 0.001
Question (3)—Straightening	0.351	< 0.001
Question (4)—Going up or down	0.370	< 0.001
Question (5)—Standing	0.395	< 0.001
Question (6)—Rising	0.406	< 0.001
Question (7)—Bending	0.392	< 0.001
PROMIS-10^††^		
Global physical health T-score	−0.161	< 0.001
Global mental health T-score	0.024	0.433
Global01—General health	0.024	0.434
Global02—General quality of life	−0.027	0.381
Global03—General physical health	−0.003	0.931
Global04—General mental health	0.084	0.006
Global05—General satisfaction on social activities	−0.013	0.671
Global09—General carrying out of social activities	−0.112	< 0.001
Global06—Carrying out of physical activities	−0.135	< 0.001
Global10—Emotional problems	0.007	0.809
Global08—Fatigue	−0.104	0.001
Global07—Pain	−0.266	< 0.001

^†^Questions: How severe is your knee stiffness after (1) first wakening in the morning, (2) twisting or pivoting on your knee, (3) straightening your knee fully, (4) going up or down stairs, (5) standing upright, (6) rising from sitting, and (7) bending to the floor to pick up an object

^††^Global01: In general, how would you say your health is?; Global02: In general, how would you say your quality of life is?; Global03: In general, how would you rate your physical health?; Global04: In general, how would you rate your mental health, including your mood and your ability to think?; Global05: In general, how would you rate your satisfaction with your social activities and relationships?; Global06: To what extent are you able to carry out your everyday physical activities?; Global07: How would you rate your pain on average?; Global08: How would you rate your fatigue on average?; Global09: In general, please rate how well you carry out your usual social activities and roles; Global10: How often have you been bothered by emotional problems such as feeling anxious, depressed, or irritable?

*KOOS JR* Knee Disability and Osteoarthritis Outcome Score Joint Replacement, *PROMIS-10* Patient-Reported Outcomes Measurement Information System-10

**Fig. 2:**
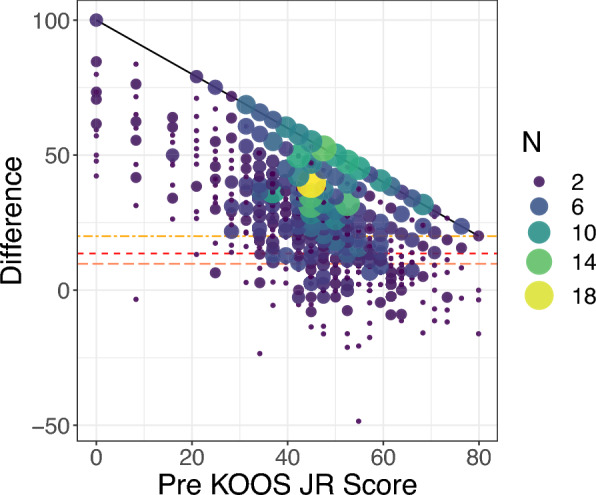
Relationships between the change in KOOS JR scores and the preoperative KOOS JR score. Changes below the three different horizontal lines are considered a failure to achieve the corresponding outcomes: the distribution-based MCID (the bottom horizontal line), the anchor-based MCID (middle), and the SCB (top). Diagonal (asymptotic) line indicates the maximum improvement score given a preoperative score. *KOOS JR* Knee Disability and Osteoarthritis Outcome Score Joint Replacement, *MCID* minimal clinically important difference, *SCB* substantial clinical benefit

Regarding threshold-based outcomes, the majority of patients (*N* = 745, 70.0%) achieved all three clinical score thresholds (Fig. 2), with an average change of 30.0 points (first quartile = 16.3; third quartile = 42.2). A small number of patients (*N* = 49, 4.6%) reported worse scores postoperatively compared with their preoperative scores on the KOOS JR (i.e., change score was negative). Furthermore, the delta and the preoperative score formed a moderately negative correlation (*r* = −0.530, *P* < 0.001), and those with a lower preoperative score were more likely to exceed the three outcomes. In addition, 159 patients (14.9%) reached the maximum improvement possible following the TKA, with a postoperative score of 100. Of 1064 patients, 145 (13.6%) did not achieve the distribution-based MCID, 216 (20.3%) did not achieve the anchor-based MCID, and 319 (30.0%) patients did not achieve the SCB.

**Fig. 3:**
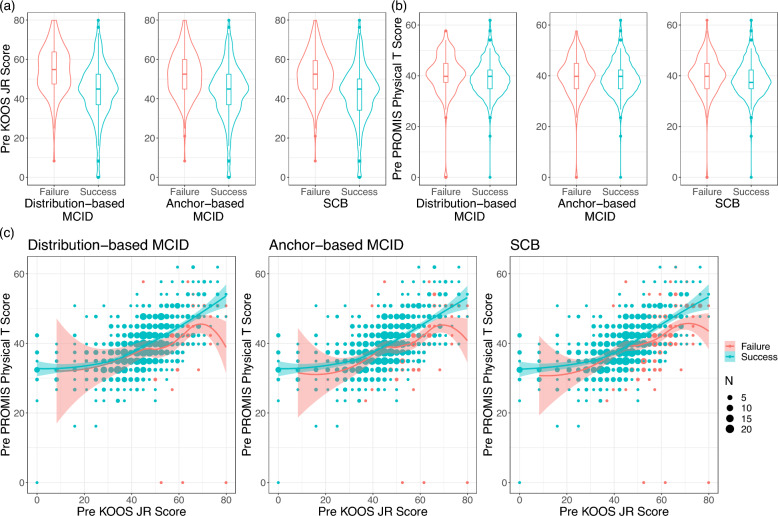
Relationships between the preoperative scores and the threshold-based outcomes. **a** Relationships between the preoperative KOOS JR score and the threshold-based outcomes. **b** Relationships between the preoperative PROMIS-10 global physical T-score and the threshold-based outcomes. **c** Relationships between the preoperative PROMIS-10 global physical T-score and the preoperative KOOS JR score across the threshold-based outcomes. *KOOS JR* Knee Disability and Osteoarthritis Outcome Score Joint Replacement, *MCID* minimal clinically important difference, *PROMIS-10* Patient-Reported Outcomes Measurement Information System-10, *SCB* substantial clinical benefit

We further examined the relationships between the threshold-based outcomes and survey responses on general health and knee-specific function. Figure 3a shows that patients who reached each threshold tended to have a statistically significantly lower preoperative KOOS JR score (*P* < 0.001) than those who did not attain each achievement. In contrast, little to no difference existed in the PROMIS-10 global physical health T-scores between patients who met the thresholds and those who did not (i.e., distribution- and anchor-based MCID: *P* = 0.21 and 0.38; SCB: *P* = 0.002, Fig. 3b). However, when two preoperative scores were visualized together, as shown in Fig. 3c, those with a higher preoperative PROMIS-10 global physical health T-score when conditioning on the same preoperative KOOS JR score were more likely to achieve all the outcomes. Among patients who were top performers on the KOOS JR scores (i.e., KOOS JR ≥ 52.5; *N* = 349), those with high PROMIS-10 global physical health T-scores (i.e., PROMIS-10 ≥ 44.9; *N* = 179) were more likely to exceed the thresholds than those with low T-scores (PROMIS-10 < 44.9; *N* = 170): 78.2% versus 71.8% for the distribution-based MCID, 72.1% versus 58.8% for the anchor-based MCID, and 58.1% versus 43.5% for the SCB.

### Predictive analyses

For evaluation purposes, five separate models were built using the entire covariate set. All method performances in terms of the AUC and F1 score for the three threshold-based outcomes (i.e., distribution-based MCID, anchor-based MCID, and SCB) are presented in Table 2. Both the PLR and RF models were identified to produce close to or exceed the 0.70 AUC, which is considered as acceptable AUC in literature [47, 62], for all the three outcomes (i.e., averages [95% CI] for distribution-based MCID = 0.708 [0.698–0.719]; anchor-based MCID = 0.691–0.706 [0.683–0.713]; SCB = 0.713–0.720 [0.706–0.726]). However, as presented in Supplementary Table S2, RF models suffered from overfitting, meaning they performed well on the training set but significantly worse on the test set. Furthermore, F1 scores from the PLR models surpassed those from the others and the baseline for all the outcomes (averages [95% CI] for distribution-based MCID = 0.348 [0.336–0.361]; anchor-based MCID = 0.412 [0.400–0.423]; SCB = 0.526 [0.516–0.536]). Supplementary Table S3 presents the recall and precision performance of the models, and the recall exceeded the precision across most cases.

**Table 2:** Model performances

Methods	Distribution-based MCID		Anchor-based MCID		SCB	
	AUC (avg [95% CI])	F1 (avg [95% CI])	AUC (avg [95% CI])	F1 (avg [95% CI])	AUC (avg [95% CI])	F1 (avg [95% CI])
Baseline	0.500 (0.500–0.500)	0.209 (0.200–0.218)	0.500 (0.500–0.500)	0.285 (0.276–0.294)	0.500 (0.500–0.500)	0.368 (0.359–0.377)
Penalized logistic regression	0.708 (0.698–0.719)	0.348 (0.336–0.361)	0.691 (0.683–0.699)	0.412 (0.400–0.423)	0.713 (0.706–0.719)	0.526 (0.516–0.536)
SVM with a polynomial kernel	0.667 (0.656–0.677)	0.306 (0.296–0.317)	0.678 (0.669–0.686)	0.399 (0.390–0.409)	0.692 (0.684–0.699)	0.513 (0.504–0.521)
SVM with a radial kernel	0.658 (0.648–0.668)	0.301 (0.290–0.312)	0.670 (0.662–0.679)	0.392 (0.382–0.403)	0.701 (0.694–0.708)	0.521 (0.513–0.529)
Random forest	0.708 (0.698–0.717)	0.324 (0.309–0.338)	0.706 (0.698–0.713)	0.397 (0.384–0.409)	0.720 (0.713–0.726)	0.523 (0.514–0.533)
XGBoost	0.706 (0.696–0.716)	0.308 (0.293–0.323)	0.693 (0.685–0.700)	0.372 (0.359–0.385)	0.691 (0.684–0.698)	0.486 (0.476–0.497)

*AUC* area under the receiver operating characteristic curve, *avg* average, *CI* confidence interval, *MCID* minimal clinically important difference, *SCB* substantial clinical benefit, *SVM* support vector machine, *XGBoost* extreme gradient boosting

**Fig. 4:**
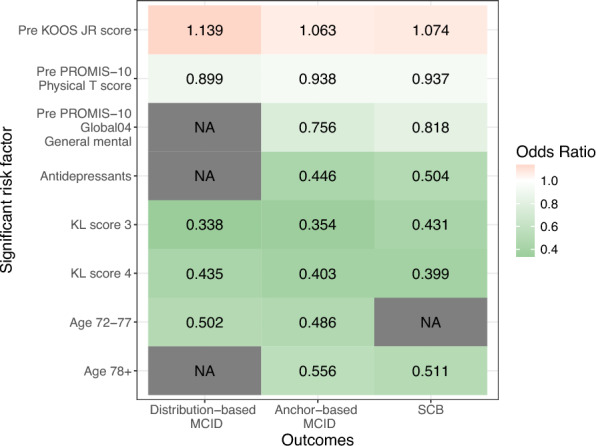
Shared risk factors across the three threshold-based outcomes. An odds ratio > 1 indicates that the factor is associated with a higher likelihood of not achieving the outcome, while an odds ratio < 1 suggests a higher likelihood of achieving the outcome. For instance, patients with a high preoperative KOOS JR score are more likely to not achieve the MCDs and the SCB, whereas those with a high preoperative PROMIS-10 global physical T-score are more likely to achieve the outcomes. *KL score* Kellgren–Lawrence score, *KOOS JR* Knee Disability and Osteoarthritis Outcome Score Joint Replacement, *MCID* minimal clinically important difference, *PROMIS-10* Patient-Reported Outcomes Measurement Information System-10, *SCB* substantial clinical benefit

We identified risk factors for the MCIDs and the SCB using logistic regression with a stepwise variable selection. These significant risk factors for the three PROMs, along with risk factors for the delta, were obtained from a linear regression model and are shown in Fig. 4 and presented in Supplementary Tables S4–S7. Risk factors selected were for either (1) all the three threshold-based outcomes or (2) any two threshold-based outcomes and the delta. As such, a total of six shared risk factors were identified; three were specific to PROMs (i.e., pre-KOOS JR, pre-PROMIS-10 physical T-score, pre-PROMIS-10 mental T-score), one specific to medication (i.e., antidepressants), one specific to demographics (i.e., age), and one specific to preoperative care (i.e., KL grade).

In the preoperative PROM category, a higher preoperative KOOS JR score was associated with poorer outcomes (ORs = 1.063–1.139; 95% confidence interval [CI] 1.034–1.179). In contrast, higher PROMIS-10 global physical health T-scores (ORs = 0.899–0.938; 95% CI 0.852–0.974) and better responses on the individual PROMIS-10 questionnaire item “In general, how would you rate your mental health, including your mood and your ability to think?” (ORs = 0.756–0.818; 95% CI 0.612–0.989) were positively associated with the achievements of the MCIDs and the SCB. These findings are consistent with the results shown in Fig. 3c.

Of the variables collected within the demographics, medication, and preoperative care categories, a higher KL grade was linked to better threshold-based outcomes (ORs = 0.338–0.435; 95% CI 0.205–0.756). Moreover, patients who had been prescribed antidepressants were more likely to achieve the anchor-based MCID and SCB (ORs = 0.446–0.504; 95% CI 0.216–0.960). Finally, the older age group of patients (i.e., 72 years and older), despite having a higher preoperative KOOS JR score (i.e., 72–77 years = 46.4 points and ≥ 78 years = 47.9 points), were more likely to achieve the MCIDs and the SCB (ORs = 0.486–0.556; 95% CI 0.288–0.903; Supplementary Tables S3–S5).

## Discussion

### Prediction of not achieving the threshold-based outcomes

Understanding the ability of patients undergoing TKA to achieve meaningful changes in disease-specific PROMs is growing ever more important for clinicians as well as policymakers, while PROM performance measures increase in importance for shared decision-making [63], quality reporting [64], and reimbursement models [65, 66]. Upon the descriptive analyses and identification of the reliable machine-learning models, the reliable preoperative identification for patients at high risk for not meeting the threshold-based outcomes was enabled (i.e., distribution-based and anchor-based MCID and the SCB). Such identification can create opportunities for improved shared decision-making preoperatively and improve risk stratification and risk adjustment for those charged with adjudicating quality of care processes for surgeons performing TKA (e.g., CMS, commercial payers, and quality-assessing organizations) [47, 62].

### Risk factors beyond preoperative KOOS JR score

The current prediction models demonstrate that patients who reported having a “better functioning” knee prior to surgery, as indicated by an overall higher score on the KOOS JR, were more likely to not meet the MCIDs, which aligns with prior studies [28, 33, 34, 67–69], and moreover, the SCB. The current study, however, provides further depth on the importance of preoperative PROMs (i.e., not just the KOOS JR score independently) to add power for identifying unique patient cohorts with different risk levels for poor outcomes following a TKA. In the current descriptive analysis (Fig. 3a–c), we identified the relationships between the changes in the total KOOS JR score and several individual survey items in both the PROMIS-10 and KOOS JR (Table 1). Furthermore, the current prediction model identified that patients who reported overall good general physical health (i.e., a high preoperative PROMIS-10 global physical T-score) but poor knee function (i.e., a low preoperative KOOS JR score) were more likely to achieve the two MCIDs and the SCB.

Moreover, using a combination of PROMs, demographics, comorbidities, and medical histories collected preoperatively, rather than relying solely on the KOOS JR score, gains a holistic view of a patient’s health state and risk factors. Our models identified that older patients were more likely to succeed in achievement of the TKA-related MCIDs and SCB when compared with younger patients; these findings are consistent with previous reported findings of patients aged 55–90 years having reported overall better outcomes postoperatively compared with those aged 19–54 years [70]. Though older patients here reported relatively high preoperative KOOS JR scores, relying solely on these scores for decision-making may misleadingly suggest a higher likelihood of not achieving the MCIDs and SCB in this population.

In addition, our predictive models demonstrated that the state of mental health preoperatively, as measured by antidepressant prescription history and PROMIS-10 mental health T-score, was significantly linked to achieving the MCIDs and the SCB cutoff values. Specifically, patients who were previously prescribed antidepressants, rather than those merely diagnosed with depression, were more likely to reach both MCIDs and SCB cutoffs. While depression history was excluded during variable selection, antidepressant prescription history remained and was considered a significant finding. In addition, patients with a high general mental health measured by PROMIS-10 Global04 (“In general, how would you rate your mental health, including your mood and your ability to think?”) were likely to surpass the threshold-based outcomes, which is also consistent in previous studies [71, 72]. Notably, patients’ self-reported mental health offered a valuable patient perspective that antidepressant prescription history alone may not capture, as both factors were retained in our final models and were considered a strong predictor.

Lastly, our model uniquely found that patients with more severe knee osteoarthritis (i.e., as indicated by a higher KL score) were more likely to achieve both MCIDs and SCB. These findings are supported by previous literature in which authors demonstrated that a more severe osteoarthritis (OA) diagnosis preoperatively often results in better pain and functional outcomes following a TKA [73, 74]. Specifically, these findings have been revealed in several studies reporting patients who were diagnosed with more severe OA reported lower preoperative scores on the PROMs and also demonstrated a larger, more significant change in scores preoperatively to postoperatively following a TKA [75]. As such, it is imperative for clinicians to consider all these preoperative patient-specific factors to ensure a more individualized approach to treatment to produce meaningful outcomes measured through the patient perspective.

### Model evaluations

Linear models, such as PLR, assume linear relationships between dependent and independent variables, leading to straightforward models and easily interpretable results [56]. Nonlinear models (e.g., SVMs with polynomial and radial kernels) can capture complex relationships between variables but are less interpretable than linear models [44]. Nonparametric models (e.g., random forest and XGBoost) do not rely on any assumption for a specific relationship and are more flexible [76]. However, their results can be more challenging to interpret compared with parametric models, which use parameters to represent these relationships. Compared with linear models, the other two models have a higher risk of overfitting, meaning that they perform well on the training set but worse on the test set, especially when the sample size is small [52]. In our prediction analysis, accounting for the 95% CI of performance metrics, the RF and the PLR demonstrated comparable and superior AUC scores on the test sets compared with the SVMs and the XGBoost. However, considering the potential for overfitting and F1 scores, the PLR could be more robust than the RF.

For the anchor-based MCID, models identified in this study can be compared with models in existing studies [33, 35]; previous authors identified success rates of achieving the MCID ranging from 76% to 82%, which are similar to that identified in the current study (i.e., 80%). The AUCs presented here are also comparable to, or even better than, those in the existing studies (i.e., average AUCs = 0.610–0.760) considering the SD [33, 35]. For the other threshold-based outcomes, a previous study analyzed the distribution-based MCID but used a different short-form version of the KOOS (i.e., Knee Injury and Osteoarthritis Outcome Score–Physical Function Shortform [KOOS-PS]) and not KOOS JR used in this study [34]. However, to our knowledge, minimal literature exists attempting to predict achieving the SCB within the arthroplasty population. Nonetheless, the current models show significant improvements in the AUC for all the three outcomes, increasing up to 46% compared with the baseline AUC. In addition, the F1 scores saw substantial gains, which increased by 70% for the distribution-based MCID, 48% for the anchor-based MCID, and 47% for the SCB, compared with the respective baseline F1 scores. In scenarios where one class predominates, the F1 score tends to be lower owing to the scarcity of positive cases, as seen in the distribution-based MCID. Despite the challenge posed by the challenge of extreme class imbalance, the current predictive models enhanced the prediction of failures, particularly in achieving the distribution-based MCID. Notably, recall performance exceeds precision (Supplementary Table S3), indicating that the models are effective at identifying high-risk patients, albeit with a tendency to occasionally classify low-risk individuals as high risk. This false positive result in the current study may warrant a more in-depth discussion with patients as part of a shared decision-making process and should not negatively impact the care episode. Overall, given acceptable AUCs, our models demonstrate strong performance in identifying at-risk patients across both MCIDs and SCB, with a general inclination to classify patients as positive. This predictive capability can support clinical decision-making based on PROMs.

### Limitations

This study is not without limitations that warrant discussion. This study was conducted at a single academic medical center, which may limit the generalizability of the findings and make it challenging to apply them to other settings. Owing to privacy concerns, obtaining data from other institutions is challenging. Consequently, external validation is difficult, and studies on PROMs, including the current study, are primarily single-institution studies [28, 33–35]. We believe meta-analyses combining research findings have the potential to provide more comprehensive conclusions in the future [77].

Moreover, Black patients in this study (11.2%) were less represented than those in the national population (15.4%) [78]. However, bias was not identified in the outcomes within the current data across racial groups (Supplementary Table S8). In addition, the racial factor was not identified as significant, demonstrating that there were no racial differences in achieving the threshold-based outcomes. Nonetheless, future research should collect and incorporate a larger sample from minority groups to minimize the risk of algorithmic bias.

Furthermore, this study did not collect responses to an anchor question from our population and adopted established thresholds for the anchor-based MCID and SCB. Although these thresholds may not be applicable to our study population, the rates at which patients achieved the anchor-based MCID and SCB were similar to other studies [28, 33, 35]. Moreover, for some patients, the absence of a 1-year postoperative survey may be associated with poorer health outcomes (i.e., missing not at random) [79], potentially leading to attrition bias [80].

Lastly, although the machine-learning models identified in the present study are comparable to those in recently published studies [33, 35], the prediction performances are generally only moderate. To improve the capability to distinguish patients, future effort should be devoted to collect more high-quality PROM data and include more preoperative predictors.

## Conclusions

This study demonstrates that machine-learning models can predict the achievement of the distribution-based MCID, the anchor-based MCID, and the SCB for TKA procedures using several preoperatively available variables, including those easily obtained through administration of the KOOS JR and PROMIS-10. The PROMIS-10 global physical health T-score and patient age affect the accuracy of the KOOS JR score in predicting score change, which may impact both the surgical decision-making process and post hoc outcome assessment. A tool that incorporates these elements for shared decision-making can provide clinicians with an evidence-based method to counsel patients considering TKA, ultimately improving care delivery and outcomes in this highly utilized intervention.

## Supplementary Information

Supplementary material 1.

## Data Availability

The datasets analyzed during the current study are available from the corresponding author on reasonable request.

## Abbreviations

ANOVA: Analysis of variance
ASA: American Society of Anesthesiologists
BMI: Body mass index
CCI: Charlson Comorbidity Index
CMS: Centers for Medicare and Medicaid Services
EHR: Electronic health record
KL score: Kellgren–Lawrence score
KOOS JR: Knee disability and osteoarthritis outcome score joint replacement
LASSO: Least absolute shrinkage and selection operator
MCID: Minimal clinically important difference
OR: Odds ratio
PROM: Patient-reported outcome measure
PROMIS-10: Patient-Reported Outcomes Measurement Information System-10
RF: Random forest
AUC: Area under the receiver operating characteristic curve
SCB: Substantial clinical benefit
SD: Standard deviation
SVM: Support vector machine
TKA: Total knee arthroplasty
XGBoost: Extreme gradient boosting
