# Supplementary material for: Preoperatively predicting failure to achieve the minimum clinically important difference and substantial clinical benefit for total knee arthroplasty patients using machine learning
Source: Knee Surg Relat Res. 2025 Sep 10;37:37. doi: 10.1186/s43019-025-00289-y (PMC12424216; doi:10.1186/s43019-025-00289-y)
Supplement: Supplementary file 1 — Supplementary material 1. [file 43019_2025_289_MOESM1_ESM.docx]

Supplementary Material

**S.1 Covariates**

In this study, we included the following variable categories: 1) demographic (e.g., age, sex) and socioeconomic status, 2) preoperative care (e.g., body mass index [BMI], Charlson Comorbidity Index [CCI]), 3) comorbidities and medical histories documented within 90 days of the index procedure, 4) medications documented within 90 days of the index procedure, and 5) preoperative survey. Variables regarding preoperative survey are listed in Table 1, and the distributions of the remaining variables are exhibited in Table S1.

Table S1 The distributions of the covariates. Abbreviations: ASA, American Society of Anesthesiologists; BMI, body mass index; COPD, Chronic obstructive pulmonary disease, CVA, cerebrovascular accident; DVT, deep vein thrombosis; Dx, diagnosis; Hx, medical history; KL score, Kellgren-Lawrence score; KOOS JR, knee injury disability and osteoarthritis outcome score joint replacement; MCID, minimal clinically important difference; Misc, miscellaneous; NSAID, non-steroidal anti-inflammatory drugs; PROMIS, patient-reported outcomes measurement information system; PTSD, post-traumatic stress disorder; TIA, transient ischemic attack; Tx, treatment.

| Covariates | Levels | N (%) | Mean (Q1, Q3) of or Correlation with Pre KOOS JR Scores | p-value |
| --- | --- | --- | --- | --- |
| **Demographics and Socioeconomic status** | | | | |
| Sex | Female | 659 (61.9) | 30.3 (16.5, 42.3) | 0.553 |
|  | Male | 405 (38.1) | 29.5 (16.3, 40.6) |  |
| Age | 16 - 66 | 265 (24.9) | 29.1 (15.9, 43.0) | 0.808 |
|  | 67 - 71 | 224 (21.1) | 29.9 (16.2, 40.7) |  |
|  | 72 - 77 | 301 (28.3) | 30.2 (18.2, 40.6) |  |
|  | 78+ | 274 (25.8) | 30.7 (16.5, 45.0) |  |
| Race | White | 679 (63.8) | 30.5 (16.9, 42.2) | 0.492 |
|  | Black | 86 (8.1) | 30.0 (16.6, 45.6) |  |
|  | Other | 299 (28.1) | 28.9 (15.8, 41.1) |  |
| Marital Status | Single | 164 (15.4) | 29.5 (17.1, 42.5) | 0.613 |
|  | Married | 653 (61.4) | 29.6 (16.0, 40.6) |  |
|  | Divorced/Separated/Widowed | 159 (14.9) | 31.5 (18.5, 45.2) |  |
|  | Other or Unknown | 88 (8.3) | 31.3 (17.4, 43.3) |  |
| Insurance | Private or Managed care | 265 (24.9) | 30.2 (18.2, 40.3) | 0.409 |
|  | Medicare or Medicaid | 763 (71.7) | 30.1 (16.2, 42.3) |  |
|  | Others | 36 (3.4) | 25.8 (12.8, 40.8) |  |
| Missing indicator of SES | No | 145 (13.6) | 27.5 (13.5, 41.7) | 0.119 |
|  | Missing | 919 (86.4) | 30.4 (16.9, 42.2) |  |
| **Preoperative care** | | | | |
| Weight at admission |  |  | 0.072 | 0.019 |
| BMI at admission | <25.0 | 304 (28.6) | 29.7 (16.6, 42.3) | 0.240 |
|  | 25.0 - 29.9 | 116 (10.9) | 28.3 (15.3, 39.9) |  |
|  | 30.0 - 39.9 | 488 (45.9) | 30.5 (16.9, 44.5) |  |
|  | ≥ 40.0 | 156 (14.7) | 32.0 (20.8, 42.1) |  |
| Radiation Hx 3 month prior | N | 119 (11.2) | 29.8 (18.1, 40.4) | 0.918 |
|  | Y | 945 (88.8) | 30.0 (16.3, 42.3) |  |
| Charlson Comorbidity Index | 0 | 943 (88.6) | 29.7 (16.3, 40.6) | 0.081 |
|  | 1 | 68 (6.4) | 29.9 (15.2, 46.8) |  |
|  | 2+ | 53 (5.0) | 35.8 (21.8, 50.0) |  |
| ASA Classification | 1 or 2 | 371 (34.9) | 28.3 (16.5, 40.6) | 0.034 |
|  | 3 or 4 | 693 (65.1) | 30.9 (16.3, 43.0) |  |
| KL Score | 1 or 2 | 174 (16.4) | 25.1 (11.5, 37.1) | 0.000 |
|  | 3 | 590 (55.5) | 30.3 (18.3, 40.6) |  |
|  | 4 | 300 (28.2) | 32.2 (18.0, 47.5) |  |
| **Medical history** | | | | |
| Flu Vaccine Hx | N | 520 (48.9) | 30.2 (16.1, 42.5) | 0.774 |
|  | Y | 544 (51.1) | 29.8 (16.9, 40.7) |  |
| Depression | N | 1007 (94.6) | 30.0 (16.2, 42.3) | 0.993 |
|  | Y | 57 (5.4) | 30.0 (20.8, 39.4) |  |
| Anxiety | N | 666 (62.6) | 30.5 (16.9, 42.2) | 0.281 |
|  | Y | 398 (37.4) | 29.2 (16.1, 41.9) |  |
| Bipolar disorders | N | 1042 (97.9) | 30.0 (16.7, 42.0) | 0.716 |
|  | Y | 22 (2.1) | 31.9 (10.8, 48.2) |  |
| Behavioral disorders | N | 583 (54.8) | 30.3 (16.9, 42.0) | 0.529 |
|  | Y | 481 (45.2) | 29.6 (16.1, 42.3) |  |
| Severe Stress or PTSD | N | 1014 (95.3) | 30.0 (16.3, 42.1) | 0.895 |
|  | Y | 50 (4.7) | 30.3 (17.4, 43.4) |  |
| Parkinsons | N | 1040 (97.7) | 29.9 (16.3, 42.0) | 0.171 |
|  | Y | 24 (2.3) | 35.4 (26.4, 53.8) |  |
| Liver disease | N | 965 (90.7) | 29.6 (16.0, 42.0) | 0.027 |
|  | Y | 99 (9.3) | 34.1 (21.8, 46.0) |  |
| Anemia | N | 889 (83.6) | 29.7 (16.3, 42.0) | 0.238 |
|  | Y | 175 (16.4) | 31.7 (16.4, 46.0) |  |
| Osteoporosis | N | 887 (83.4) | 29.6 (16.2, 42.0) | 0.145 |
|  | Y | 177 (16.6) | 31.9 (16.9, 45.0) |  |
| Obesity | N | 382 (35.9) | 28.9 (15.9, 40.6) | 0.15 |
|  | Y | 682 (64.1) | 30.6 (17.1, 42.3) |  |
| Solid Organ Transplant | N | 1041 (97.8) | 29.9 (16.2, 42.0) | 0.096 |
|  | Y | 23 (2.2) | 36.0 (24.6, 50.2) |  |
| Chronic Pain | N | 345 (32.4) | 30.7 (18.5, 42.3) | 0.406 |
|  | Y | 719 (67.6) | 29.7 (15.8, 42.0) |  |
| Degen Knee Osteoarthritis | N | 18 (1.7) | 29.0 (21.2, 37.9) | 0.821 |
|  | Y | 1046 (98.3) | 30.0 (16.3, 42.3) |  |
| Post Traumatic Knee Osteoarthritis | N | 936 (88.0) | 30.4 (16.9, 42.3) | 0.073 |
|  | Y | 128 (12.0) | 26.9 (13.8, 39.6) |  |
| Systemic Sepsis | N | 969 (91.1) | 29.8 (16.7, 41.1) | 0.413 |
|  | Y | 95 (8.9) | 31.8 (14.5, 49.8) |  |
| Malignancy or Cancer | N | 388 (36.5) | 30.4 (17.8, 42.2) | 0.601 |
|  | Y | 676 (63.5) | 29.8 (16.2, 42.0) |  |
| Bleeding Disorder | N | 1012 (95.1) | 29.8 (16.3, 42.0) | 0.155 |
|  | Y | 52 (4.9) | 34.3 (21.6, 52.5) |  |
| Endocrine | N | 746 (70.1) | 30.2 (18.3, 41.9) | 0.598 |
|  | Y | 318 (29.9) | 29.5 (13.6, 43.0) |  |
| Any Diabetes | N | 738 (69.4) | 30.0 (17.9, 41.6) | 0.956 |
|  | Y | 326 (30.6) | 29.9 (15.1, 44.5) |  |
| Mood Disorder | N | 989 (93.0) | 30.0 (16.7, 42.2) | 0.976 |
|  | Y | 75 (7.0) | 30.1 (15.1, 40.8) |  |
| Sleep Apnea | N | 398 (37.4) | 29.6 (16.2, 41.9) | 0.588 |
|  | Y | 666 (62.6) | 30.2 (16.9, 42.3) |  |
| Atrial Fibrillation | N | 865 (81.3) | 30.0 (16.3, 42.3) | 0.901 |
|  | Y | 199 (18.7) | 29.8 (16.6, 39.7) |  |
| Ventricular Arrhythmia | N | 892 (83.8) | 29.5 (16.2, 41.3) | 0.073 |
|  | Y | 172 (16.2) | 32.3 (19.4, 47.5) |  |
| Congestive Heart Failure | N | 905 (85.1) | 29.7 (16.7, 41.1) | 0.224 |
|  | Y | 159 (14.9) | 31.9 (15.9, 47.5) |  |
| CVA or TIA | N | 831 (78.1) | 30.1 (16.9, 42.0) | 0.834 |
|  | Y | 233 (21.9) | 29.7 (15.9, 44.5) |  |
| Other Aneurysm | N | 1042 (97.9) | 29.9 (16.3, 42.0) | 0.374 |
|  | Y | 22 (2.1) | 34.3 (21.4, 49.2) |  |
| DVT | N | 961 (90.3) | 30.1 (17.1, 42.0) | 0.527 |
|  | Y | 103 (9.7) | 28.8 (13.3, 44.5) |  |
| Emphysema | N | 1017 (95.6) | 30.2 (16.9, 42.3) | 0.087 |
|  | Y | 47 (4.4) | 24.7 (10.1, 33.1) |  |
| COPD | N | 944 (88.7) | 30.4 (17.9, 42.3) | 0.042 |
|  | Y | 120 (11.3) | 26.5 (13.5, 37.2) |  |
| Asthma | N | 850 (79.9) | 29.7 (16.9, 41.1) | 0.404 |
|  | Y | 214 (20.1) | 31.0 (15.9, 45.2) |  |
| Interstitial | N | 1041 (97.8) | 30.0 (16.7, 42.2) | 0.67 |
|  | Y | 23 (2.2) | 28.1 (12.0, 39.7) |  |
| Renal Failure | N | 912 (85.7) | 29.8 (16.9, 40.6) | 0.406 |
|  | Y | 152 (14.3) | 31.3 (13.8, 48.5) |  |
| Dyspnea | N | 903 (84.9) | 29.6 (16.3, 41.7) | 0.147 |
|  | Y | 161 (15.1) | 32.2 (16.9, 47.1) |  |
| Preoperative Open Wound or Wound Infection | N | 1044 (98.1) | 29.8 (16.2, 42.0) | 0.021 |
|  | Y | 20 (1.9) | 41.3 (28.2, 53.1) |  |
| Insulin or Diabetes Tx | N | 982 (92.3) | 30.1 (16.9, 42.1) | 0.656 |
|  | Y | 82 (7.7) | 29.0 (15.7, 41.9) |  |
| Chronic Oxygen Use | N | 1008 (94.7) | 30.0 (16.7, 42.0) | 0.89 |
|  | Y | 56 (5.3) | 30.4 (13.8, 44.5) |  |
| Blood Transfusion without Reported Dx | N | 945 (88.8) | 29.8 (16.7, 42.0) | 0.524 |
|  | Y | 119 (11.2) | 31.2 (15.8, 47.3) |  |
| Coronary Artery Disease | N | 731 (68.7) | 29.6 (16.2, 42.2) | 0.271 |
|  | Y | 333 (31.3) | 30.9 (18.3, 42.0) |  |
| Hypertension | N | 176 (16.5) | 28.8 (16.1, 39.9) | 0.371 |
|  | Y | 888 (83.5) | 30.2 (16.6, 42.3) |  |
| Valvular Disease | N | 885 (83.2) | 29.8 (16.3, 42.0) | 0.487 |
|  | Y | 179 (16.8) | 30.9 (16.6, 44.0) |  |
| Missing indicator of Medical Hx | N | 1020 (95.9) | 29.9 (16.3, 42.0) | 0.448 |
|  | Y | 44 (4.1) | 32.4 (15.3, 47.6) |  |
| **Medication** | | | | |
| Narcotic Analgesics | N | 774 (72.7) | 30.2 (17.1, 42.3) | 0.657 |
|  | Y | 290 (27.3) | 29.6 (15.8, 40.3) |  |
| Nonnarcotic Analgesics | N | 872 (82.0) | 30.5 (18.3, 42.3) | 0.053 |
|  | Y | 192 (18.0) | 27.5 (14.1, 40.6) |  |
| Antianxiety Agents | N | 1029 (96.7) | 29.8 (16.7, 42.0) | 0.167 |
|  | Y | 35 (3.3) | 34.9 (15.8, 47.6) |  |
| Antidepressants | N | 983 (92.4) | 29.8 (16.2, 42.0) | 0.152 |
|  | Y | 81 (7.6) | 32.9 (21.5, 42.9) |  |
| Corticosteroids | N | 996 (93.6) | 30.3 (16.9, 42.3) | 0.108 |
|  | Y | 68 (6.4) | 26.0 (14.1, 34.9) |  |
| Hypnotics | N | 1020 (95.9) | 30.0 (16.3, 42.0) | 0.786 |
|  | Y | 44 (4.1) | 29.2 (15.8, 43.5) |  |
| Psychotherapeutic - Misc. | N | 1032 (97.0) | 30.3 (16.7, 42.3) | 0.006 |
|  | Y | 32 (3.0) | 21.6 (12.5, 32.4) |  |

**S.2 Model evaluation**

Table S2 AUC performances for training and test sets.

|  | Distribution-based MCID | | Anchor-based MCID | | SCB | |
| --- | --- | --- | --- | --- | --- | --- |
|  | Training (avg [**95% CI**]) | Test (avg [**95% CI**]) | Training (avg [**95% CI**]) | Test (avg [**95% CI**]) | Training (avg [**95% CI**]) | Test (avg [**95% CI**]) |
| Penalized Logistic Regression | 0.759 (0.754,0.763) | 0.708 (0.698,0.719) | 0.766 (0.761,0.770) | 0.691 (0.683,0.699) | 0.774 (0.770,0.777) | 0.713 (0.706,0.719) |
| SVM with a polynomial kernel | 0.995 (0.994,0.995) | 0.667 (0.656,0.677) | 0.982 (0.980,0.983) | 0.678 (0.669,0.686) | 0.922 (0.919,0.925) | 0.692 (0.684,0.699) |
| SVM with a radial kernel | 0.985 (0.983,0.986) | 0.658 (0.648,0.668) | 0.961 (0.958,0.963) | 0.67 (0.662,0.679) | 0.878 (0.872,0.884) | 0.701 (0.694,0.708) |
| Random Forest | 1.000 (1.000,1.000) | 0.708 (0.698,0.717) | 1.000 (1.000,1.000) | 0.706 (0.698,0.713) | 1.000 (1.000,1.000) | 0.720 (0.713,0.726) |
| **XGBoost** | **0.925 (0.918,0.931)** | **0.706 (0.696,0.716)** | **0.917 (0.909,0.926)** | **0.693 (0.685,0.700)** | **0.889 (0.880,0.898)** | **0.691 (0.684,0.698)** |

**Table S3 Recall and precision performances.**

|  | **Distribution-based MCID** | | **Anchor-based MCID** | | **SCB** | |
| --- | --- | --- | --- | --- | --- | --- |
|  | **Recall (avg [95% CI])** | **Precision (avg [95% CI])** | **Recall (avg [95% CI])** | **Precision (avg [95% CI])** | **Recall (avg [95% CI])** | **Precision (avg [95% CI])** |
| **Penalized Logistic Regression** | **0.435 (0.414,0.455)** | **0.301 (0.287,0.315)** | **0.483 (0.462,0.504)** | **0.37 (0.358,0.382)** | **0.651 (0.633,0.668)** | **0.447 (0.437,0.457)** |
| **SVM with a polynomial kernel** | **0.543 (0.523,0.564)** | **0.218 (0.208,0.227)** | **0.602 (0.583,0.621)** | **0.303 (0.294,0.312)** | **0.680 (0.667,0.694)** | **0.415 (0.406,0.425)** |
| **SVM with a radial kernel** | **0.440 (0.423,0.457)** | **0.234 (0.223,0.245)** | **0.509 (0.491,0.527)** | **0.324 (0.314,0.334)** | **0.649 (0.633,0.666)** | **0.442 (0.431,0.452)** |
| **Random Forest** | **0.367 (0.343,0.391)** | **0.313 (0.297,0.33)** | **0.481 (0.455,0.507)** | **0.356 (0.344,0.368)** | **0.627 (0.603,0.651)** | **0.464 (0.452,0.475)** |
| **XGBoost** | **0.311 (0.292,0.33)** | **0.321 (0.305,0.337)** | **0.383 (0.364,0.402)** | **0.371 (0.358,0.384)** | **0.517 (0.498,0.535)** | **0.469 (0.458,0.481)** |

**S.3 Risk factor identification**

Table S4 Logistic Regression Results for the Distribution-based MCID. An odds ratio greater than 1 indicates that the factor is associated with a higher likelihood of not achieving the outcome, while an odds ratio less than 1 suggests a higher likelihood of achieving the outcome. Abbreviations: DVT, deep vein thrombosis; Hx, medical history; KL score, Kellgren-Lawrence score; KOOS JR, Knee Disability and Osteoarthritis Outcome Score Joint Replacement; PROMIS, patient-reported outcomes measurement information system.

|  | Odds ratio | P-value |
| --- | --- | --- |
| (Intercept) | 0.008 | **<0.001** |
| **Preoperative Surveys** | | |
| KOOS JR Score | 1.139 | **<0.001** |
| PROMIS Global health T score - Physical | 0.899 | **<0.001** |
| Question (2) - Twisting | 1.343 | 0.050 |
| Question (3) - Straightening | 1.279 | 0.091 |
| Global03 - General physical health | 1.355 | **0.050** |
| Global04 - General mental health | 0.811 | 0.119 |
| Global09 - General carrying out of social activities | 1.447 | **0.003** |
| **Medical history** | | |
| Flu Vaccine Hx | 0.696 | 0.093 |
| Sleep Apnea | 1.581 | **0.045** |
| DVT | 1.716 | 0.085 |
| Interstitial | 3.482 | **0.037** |
| Renal Failure | 1.919 | **0.034** |
| Chronic Oxygen Use | 0.430 | 0.124 |
| Coronary Artery Disease | 0.711 | 0.159 |
| Valvular Disease | 0.596 | 0.097 |
| Medication |  |  |
| Antidepressants | 0.430 | 0.082 |
| **Demographics and Socioeconomic status** | | |
| Age (reference: 16 - 66) |  |  |
| 67 - 71 | 0.563 | 0.052 |
| 72 - 77 | 0.502 | **0.015** |
| 78+ | 0.619 | 0.099 |
| Missingness in socio-economic status | 0.546 | **0.031** |
| KL Score (reference: 1 or 2) |  |  |
| 3 | 0.338 | **<0.001** |
| 4 | 0.435 | **0.003** |

Table S5 Logistic Regression Results for the Anchor-based MCID. An odds ratio greater than 1 indicates that the factor is associated with a higher likelihood of not achieving the outcome, while an odds ratio less than 1 suggests a higher likelihood of achieving the outcome. Abbreviations: KL score, Kellgren-Lawrence score; KOOS JR, Knee Disability and Osteoarthritis Outcome Score Joint Replacement; PROMIS, patient-reported outcomes measurement information system.

|  | Odds ratio | P-value |
| --- | --- | --- |
| (Intercept) | 1.332 | 0.810 |
| **Preoperative Surveys** | | |
| KOOS JR Score | 1.063 | **<0.001** |
| PROMIS Global health T score - Physical | 0.938 | **0.001** |
| Question (4) - Going up or down | 0.832 | 0.135 |
| Question (6) - Rising | 0.800 | 0.112 |
| Global04 - General mental health | 0.756 | **0.009** |
| Global05 - General satisfaction on social activities | 1.276 | **0.024** |
| **Medical history** | | |
| Liver disease | 0.484 | **0.042** |
| Chronic Pain | 1.400 | 0.075 |
| Endocrine | 1.728 | **0.003** |
| Any Diabetes | 1.428 | 0.056 |
| Emphysema | 3.387 | **0.001** |
| Renal Failure | 1.852 | **0.014** |
| Preoperative Open Wound or Wound Infection | 0.226 | 0.176 |
| Valvular Disease | 0.685 | 0.134 |
| **Medication** | | |
| Antidepressants | 0.446 | **0.029** |
| **Demographics and Socioeconomic status** | | |
| Age (reference: 16 - 66) |  |  |
| 67 - 71 | 0.586 | **0.034** |
| 72 - 77 | 0.486 | **0.003** |
| 78+ | 0.556 | **0.018** |
| **Preoperative care** | | |
| KL Score (reference: 1 or 2) |  |  |
| 3 | 0.354 | **<0.001** |
| 4 | 0.403 | **<0.001** |

Table S6 Logistic Regression Results for the SCB. An odds ratio greater than 1 indicates that the factor is associated with a higher likelihood of not achieving the outcome, while an odds ratio less than 1 suggests a higher likelihood of achieving the outcome. Abbreviations: CVA, cerebrovascular accident; Hx, medical history; KL score, Kellgren-Lawrence score; KOOS JR, Knee Disability and Osteoarthritis Outcome Score Joint Replacement; PROMIS, patient-reported outcomes measurement information system; TIA, transient ischemic attack.

|  | Odds ratio | P-value |
| --- | --- | --- |
| (Intercept) | 0.280 | 0.145 |
| **Preoperative Surveys** | | |
| KOOS JR Score | 1.074 | **<0.001** |
| PROMIS Global health T score - Physical | 0.937 | **0.001** |
| Question (6) - Rising | 0.779 | **0.045** |
| Global04 - General mental health | 0.818 | **0.038** |
| Global05 - General satisfaction on social activities | 1.160 | 0.126 |
| Global07 - Pain | 1.277 | 0.070 |
| **Medical history** | | |
| Depression | 0.576 | 0.154 |
| Liver disease | 0.590 | 0.078 |
| Chronic Pain | 1.342 | 0.081 |
| Post Traumatic Knee Osteoarthritis | 1.480 | 0.092 |
| Endocrine | 1.468 | **0.021** |
| Mood Disorder | 1.781 | 0.076 |
| Ventricular Arrhythmia | 0.721 | 0.144 |
| CVA or TIA | 1.442 | 0.054 |
| Emphysema | 2.205 | **0.027** |
| Preoperative Open Wound or Wound Infection | 0.280 | 0.128 |
| Medication |  |  |
| Nonnarcotic Analgesics | 1.753 | **0.003** |
| Antidepressants | 0.504 | **0.037** |
| Corticosteroids | 2.193 | **0.009** |
| **Demographics and Socioeconomic status** | | |
| Age (reference: 16 - 66) |  |  |
| 67 - 71 | 0.736 | 0.207 |
| 72 - 77 | 0.623 | 0.066 |
| 78+ | 0.511 | **0.012** |
| Race (reference: White) |  |  |
| Black | 1.947 | **0.022** |
| Other | 1.249 | 0.199 |
| Insurance (reference: Private or Managed care) |  |  |
| Medicare or Medicaid | 1.454 | 0.085 |
| Others | 2.552 | **0.031** |
| **Preoperative care** | | |
| KL Score (reference: 1 or 2) |  |  |
| 3 | 0.431 | **<0.001** |
| 4 | 0.399 | **<0.001** |

Table S7 Regression Results for the Delta. A coefficient greater than 1 indicates that the factor is associated with a larger delta, while a coefficient less than 1 suggests a smaller delta. Abbreviations: BMI, body mass index; COPD, Chronic obstructive pulmonary disease; Dx, diagnosis; KL score, Kellgren-Lawrence score; KOOS JR, Knee Disability and Osteoarthritis Outcome Score Joint Replacement; PROMIS, patient-reported outcomes measurement information system; TIA, transient ischemic attack.

|  | Estimate | P-value |
| --- | --- | --- |
| (Intercept) | 50.652 | **0.000** |
| **Preoperative Surveys** | | |
| KOOS JR Score | -1.037 | **< 2e-16** |
| Question (2) - Twisting | -1.888 | **0.013** |
| Question (3) - Straightening | -1.988 | **0.004** |
| Global01 - General health | 1.109 | 0.130 |
| Global04 - General mental health | 1.908 | **0.002** |
| Global09 - General carrying out of social activities | 0.847 | 0.133 |
| Global08 - Fatigue | 0.958 | 0.158 |
| **Medical history** | | |
| Osteoporosis | 2.283 | 0.089 |
| Atrial Fibrillation | -2.139 | 0.114 |
| Ventricular Arrhythmia | 2.389 | 0.094 |
| COPD | -2.459 | 0.115 |
| Preoperative Open Wound or Wound Infection | 6.449 | 0.070 |
| Blood Transfusion without Reported Dx | 2.708 | 0.075 |
| **Medication** | | |
| Nonnarcotic Analgesics | -1.781 | 0.156 |
| Antidepressants | 4.328 | **0.017** |
| Corticosteroids | -4.236 | **0.034** |
| **Demographics and Socioeconomic status** | | |
| Age (reference: 16 - 66) |  |  |
| 67 - 71 | 3.004 | **0.046** |
| 72 - 77 | 4.851 | **0.002** |
| 78+ | 6.289 | **<0.001** |
| Race (reference: White) |  |  |
| Black | -5.167 | **0.005** |
| Other | -1.339 | 0.215 |
| Insurance (reference: Private or Managed care) |  |  |
| Medicare or Medicaid | -2.180 | 0.106 |
| Others | -4.508 | 0.107 |
| Missingness in socio-economic status | 3.334 | **0.019** |
| **Preoperative care** | | |
| Weight at admission | 0.052 | **0.002** |
| BMI at admission (reference: 25.0 - 29.9) |  |  |
| < 25.0 | 2.470 | 0.162 |
| 30.0 - 39.9 | -2.383 | 0.086 |
| ≥ 40.0 | -2.619 | 0.240 |
| KL Score (reference: 1 or 2) |  |  |
| 3 | 5.458 | **<0.001** |
| 4 | 7.276 | **<0.001** |

**S.4 Bias Check**

**Table S8 Failure rate comparison by racial groups. Abbreviations: KOOS JR, knee injury disability and osteoarthritis outcome score joint replacement; MCID, minimal clinically important difference.**

| **Failure rate** | **Black (N = 86)** | **White (N = 679)** |
| --- | --- | --- |
| **Distribution-based MCID** | **12.8%** | **13.3%** |
| **Anchor-based MCID** | **22.1%** | **19.4%** |
| **SCB** | **31.4%** | **28.6%** |
